# Inhibition of lanosterol synthase linking with MAPK/JNK signaling pathway suppresses endometrial cancer

**DOI:** 10.1038/s41420-025-02325-y

**Published:** 2025-02-08

**Authors:** Liangjian Ma, Wunan Huang, Xiaolei Liang, Hongli Li, Wei Yu, Lexin Liu, Yuelin Guan, Chang Liu, Xiangjun Chen, Lidan Hu

**Affiliations:** 1 The First Clinical Medical College, Lanzhou University, Lanzhou, China; 2 Gansu Provincial Clinical Research Center for Gynecological Oncology, The First Hospital of Lanzhou University, Lanzhou, China; 3 Eye Center of the Second Affiliated Hospital, Institute of Translational Medicine, School of Medicine, Zhejiang University, Hangzhou, China; 4 The Children’s Hospital, Zhejiang University School of Medicine, National Clinical Research Center for Child Health, Hangzhou, Zhejiang China; 5 Duke-NUS Medical School, Singapore, Singapore

**Keywords:** Endometrial cancer, Cancer metabolism

## Abstract

Endometrial cancer (EC) is a significant health threat to women, with recurrence after treatment posing a major challenge. While abnormal cholesterol metabolism has been implicated in EC progression, the underlying mechanisms remain unclear. In this study, we identified lanosterol synthase (LSS) as a key mediator in cholesterol metabolism associated with EC. We found that LSS is significantly upregulated in EC tissues. Functional assays revealed that LSS promotes cell proliferation and migration, inhibits apoptosis, and drives tumor growth in vivo. Mechanistically, LSS exerts dual effects by accumulating cholesterol esters, thereby enhancing EC cell growth, and activating the MAPK/JNK signaling pathway. Importantly, inhibition of LSS with the specific inhibitor Ro 48-8071 not only reduced EC cell proliferation and suppressed xenograft tumor growth but also inhibited the growth of patient-derived tumor-like cell clusters (PTCs). These findings establish LSS as a novel oncogene in EC, promoting tumor progression through MAPK/JNK signaling activation and cholesterol ester accumulation, and highlight the therapeutic potential of targeting LSS in EC treatment.

## Introduction

Endometrial cancer (EC) encompasses a range of malignant epithelial tumors originating from the endometrium [1]. It is one of the most common gynecological malignancies, particularly in high- and middle-income countries, ranking among the top three malignancies of the female reproductive system [2]. Despite significant advances in understanding the risk factors associated with EC, including obesity and estrogen exposure, the specific molecular mechanisms underlying its pathogenesis remain poorly understood [3, 4]. Current treatment strategies for EC predominantly rely on surgical intervention, yet challenges persist due to the lack of specific therapeutic targets [5]. While most EC patients have favorable outcomes, a subset with poorly differentiated tumors exhibit a high propensity for recurrence, leading to poorer prognoses. As a result, there is an urgent need to explore the molecular underpinnings of EC to identify novel biomarkers and develop targeted therapies that can improve patient management and outcomes [6].

Cholesterol metabolism has recently gained attention as a crucial factor in cancer biology, including in EC [7, 8]. Cholesterol serves as a vital component of cell membranes and as a precursor for the biosynthesis of steroid hormones, including estrogen [9]. Dysregulation of lipid metabolism, including cholesterol homeostasis, has been implicated in various aspects of tumor biology, such as energy metabolism, signal transduction, and angiogenesis [10, 11]. Targeted regulation of key genes and substances in the cholesterol biosynthesis process can inhibit the progression of EC, and cholesterol-lowering drugs such as tamoxifen can also inhibit the growth of EC cells [12, 13], suggesting that targeting key enzymes involved in cholesterol biosynthesis may offer therapeutic potential.

Lanosterol synthase (LSS) is a key rate-limiting enzyme in the cholesterol synthesis pathway and catalyzes the synthesis of lanosterol. Lanosterol is the first sterol formed after cyclization of squalene 2, 3-epoxide, which is converted to cholesterol by a series of enzymatic reactions [14]. Emerging evidence has associated LSS with the pathogenesis of several malignancies, including liver cancer and glioma, where it has been shown to activate critical signaling pathways such as mTOR and MAPK [15, 16]. Inhibition of LSS has demonstrated efficacy in suppressing tumor growth across various cancer types, including breast, prostate, pancreatic, and ovarian cancers [17, 18]. However, the role of LSS in EC remains unexplored.

In this study, we analyzed data from the Gene Expression Omnibus (GEO) database and found that LSS was significantly elevated in both low-grade and high-grade EC cancer tissues compared to normal and proliferating endometrial tissues. We further investigated the effects of LSS overexpression and inhibition on EC cell proliferation and tumor growth in vitro and in vivo, exploring the mechanistic role of the MAPK/JNK signaling pathway in this context. Additionally, we examined the impact of altered lipid metabolism, particularly cholesterol and cholesterol ester levels, in EC patients with high LSS expression. Our findings suggest that LSS plays a pivotal role in EC progression and may serve as a promising therapeutic target.

## Results

### LSS is significantly upregulated in EC

**Fig. 1:**
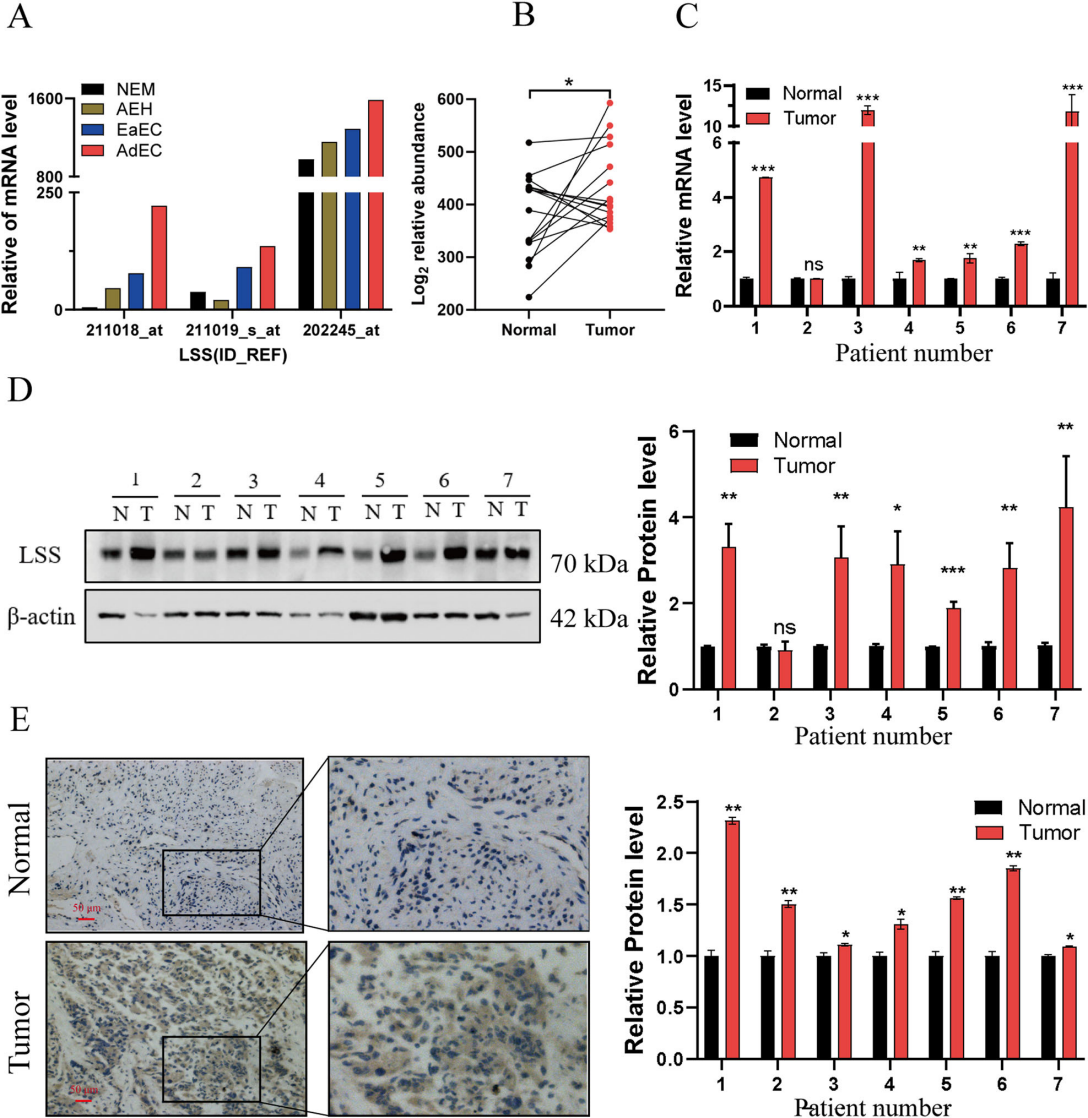
LSS is upregulated in EC tissues. **A** LSS expression in different endometrial tissues of cohort GSE39099 (10 patients per group, samples equal molar weight (10 samples/pool) combined for microarray analysis). **B** The expression of LSS protein in EC cancer tissues was measured by proteomics, and para-cancer tissues were used as control. **C** Expression of LSS mRNA in EC cancer tissue. **D** Western blotting images (left panel) and corresponding quantitative results (right panel) showing the expression of LSS in 7 paired EC samples. **E** IHC results of LSS protein expression in cancer and adjacent tissues of EC patients (×200 and ×400 magnification). NEM normal endometrial tissue, AEH endometrial hyperplasia tissue, EaEC early endometrial cancer tissues, AdEC advanced endometrial cancer tissues, N adjacent normal tissue, T tumor tissue. *p < 0.05, **p < 0.01, ***p < 0.001.

To explore the role of LSS in endometrial cancer (EC), we first analyzed its expression in the GEO dataset GSE39099. The analysis revealed that LSS expression is markedly low in normal endometrial tissues (NEM) and endometrial hyperplasia tissues (AEH), but significantly elevated in both early-stage (EaEC) and advanced-stage (AdEC) endometrial cancer tissues, with the highest levels observed in advanced-stage samples (Fig. 1A). This finding was corroborated by our previous proteomic analysis, which showed a substantial increase in LSS protein levels in EC tissues compared to adjacent non-cancerous tissues (Fig. 1B). Further validation using qPCR, Western blotting (WB), and immunohistochemistry (IHC) confirmed that LSS mRNA and protein levels were significantly higher in EC tissues compared to adjacent normal tissues (Fig. 1C–E). These data suggest that LSS is overexpressed in EC and may play a crucial role in the development and progression of the disease.

### Overexpression of LSS promotes EC cell proliferation and inhibits apoptosis in vitro

**Fig. 2:**
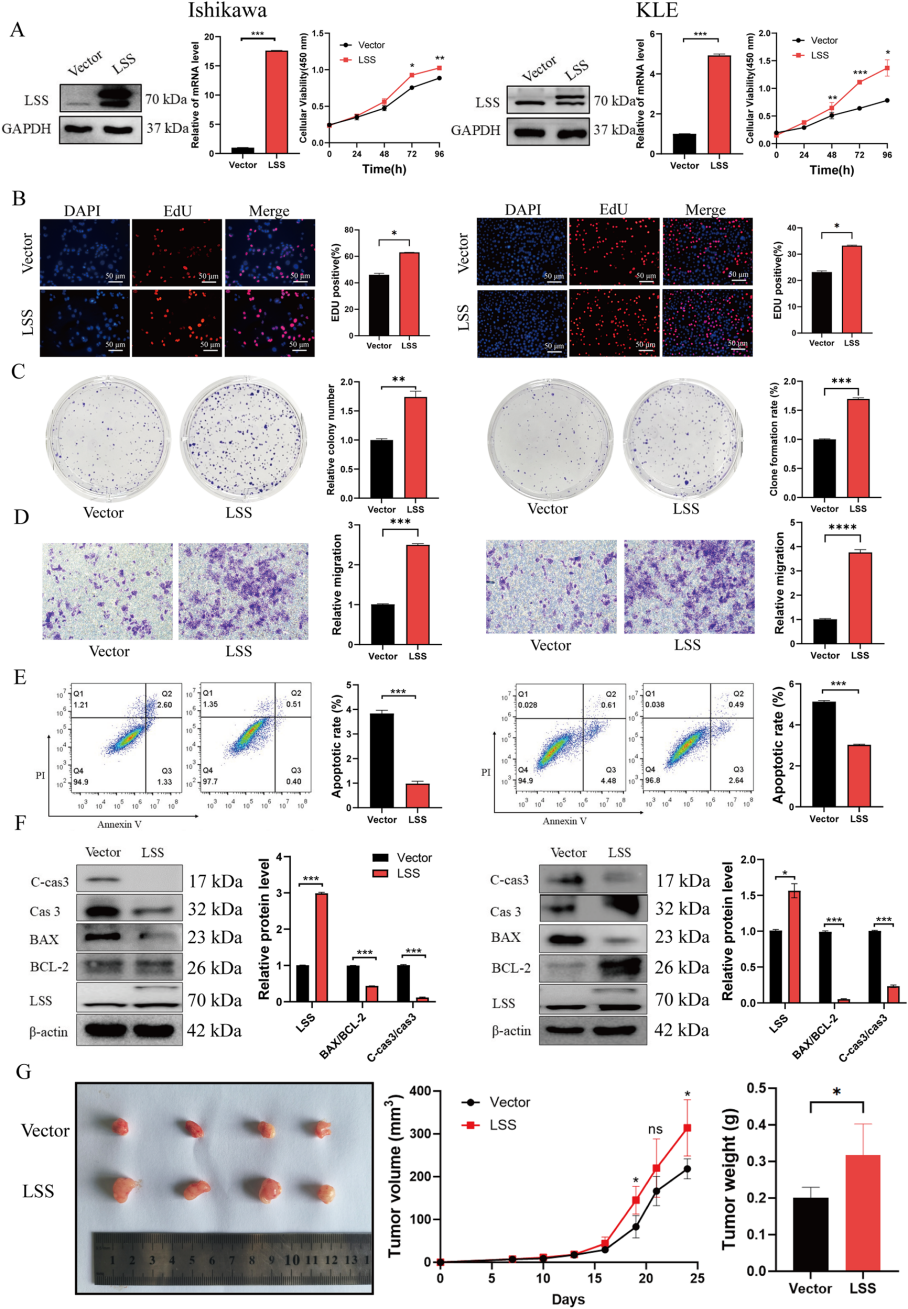
Overexpression of LSS promotes EC cell proliferation, and migration and reduces cell apoptosis. **A** qPCR and WB successfully confirmed the overexpression of LSS in EC cells, and overexpression of LSS promotes EC cell growth. **B** EdU results of LSS overexpression promoting EC cell growth. **C** The results of LSS overexpression promoting the formation of EC cell clones. **D** Overexpression of LSS promotes EC cell migration. **E**, **F** Flow cytometry and WB results of overexpression inhibiting EC cell apoptosis. **G** Images of isolated tumors from the subcutaneous tumor nude mice model established using Ishikawa cells expressing Vector or LSS overexpression, and the volume and weight of the tumor (*n* = 4 per group). *p < 0.05, **p < 0.01, ***p < 0.001.

Next, we investigated the functional impact of LSS on EC cell behavior. We assessed LSS expression in various EC cell lines and found elevated protein levels in HEC-1A, KLE, and Ishikawa cells compared to normal endometrial cells (hEEC) (Fig. S1). To further explore the role of LSS, we generated stable LSS overexpression cell lines in KLE and Ishikawa cells (Fig. 2A). Overexpression of LSS significantly enhanced the growth, EdU positivity, and colony formation of these cells (Fig. 2A–C). Additionally, LSS overexpression markedly increased the migratory capacity of EC cells (Fig. 2D). Conversely, flow cytometry analysis revealed that LSS overexpression significantly inhibited apoptosis in EC cells, as evidenced by increased expression of the anti-apoptotic protein BCL-2 and decreased levels of pro-apoptotic proteins BAX and cleaved caspase-3 (Fig. 2E, F). In vivo, nude mouse xenograft experiments demonstrated that LSS overexpression significantly promoted tumor growth (Figs. 2G and S2). Collectively, these data indicate that LSS overexpression contributes to EC cell proliferation and survival.

### Downregulation of LSS suppresses EC cell proliferation and enhances cell apoptosis

**Fig. 3:**
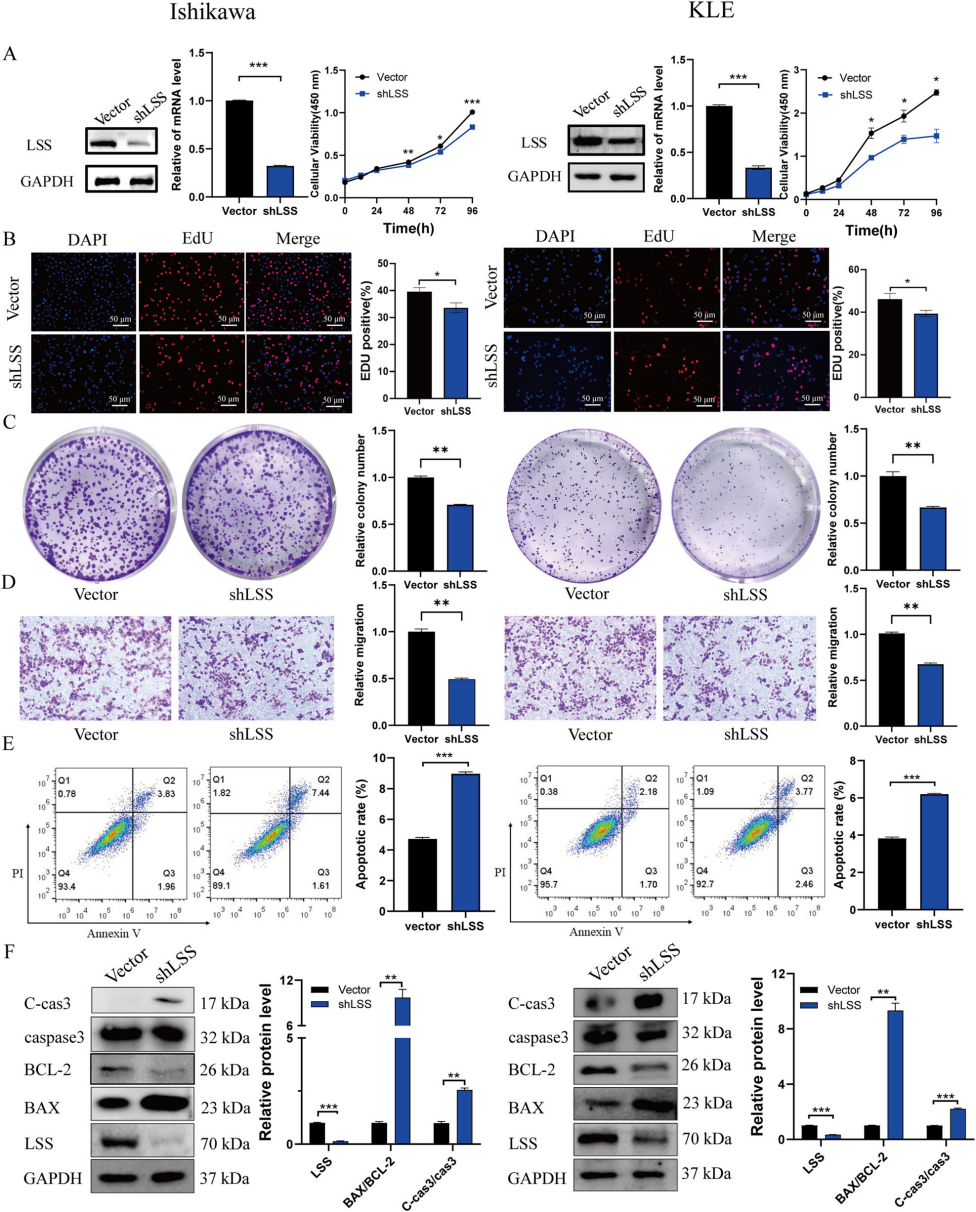
Knockdown of LSS reduces EC cell proliferation, and migration and promotes cell apoptosis. **A** qPCR and WB successfully confirmed the Knockdown of LSS in EC cells, and the Knockdown of LSS reduces EC cell growth. **B** EdU results of LSS knockdown inhibiting the growth of EC cells. **C** Results of LSS knockdown inhibiting the formation of EC cell clones. **D** LSS knockdown reduces EC cell migration. **E**, **F** Results of flow cytometry and WB detection of LSS knockdown increasing EC cell apoptosis. *p < 0.05, **p < 0.01, ***p < 0.001.

To further confirm the role of LSS in EC, we employed shRNA-mediated knockdown to reduce LSS expression in KLE and Ishikawa cell lines. Among the three LSS-targeting shRNAs tested, LSS-shRNA-A3 exhibited the highest knockdown efficiency and was selected for subsequent experiments (Fig. S3). Knockdown of LSS significantly impaired EC cell growth, reduced the EdU positivity rate, and inhibited colony formation (Fig. 3A–C). In addition, cell migration assays demonstrated that LSS knockdown markedly reduced the migratory potential of EC cells (Fig. 3D). Flow cytometry analysis showed that LSS knockdown induced apoptosis in EC cells, as evidenced by increased expression of BAX and cleaved caspase-3 and decreased levels of BCL-2 (Fig. 3E, F). These results further support the role of LSS as a critical regulator of EC cell proliferation and survival.

### LSS promotes EC cell growth through activation of the MAPK signaling pathways

**Fig. 4:**
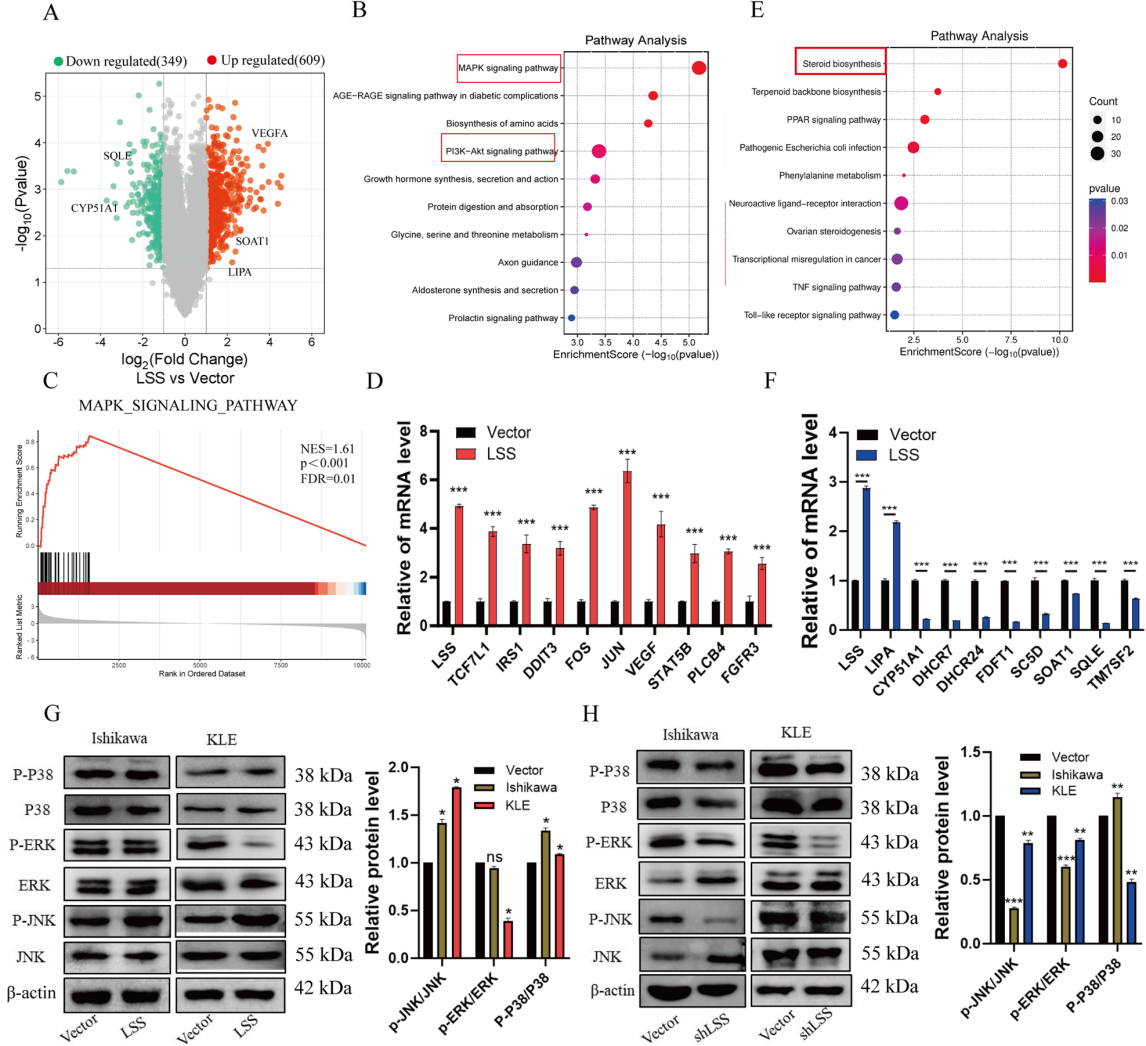
LSS promotes EC cell growth by activating downstream MAPK signaling pathways. **A** Volcano plot of differentially expressed genes after LSS overexpression. **B** Kyoto Encyclopedia of genes and genomes (KEGG) analysis showed that LSS overexpression upregulated gene enrichment pathway. **C** Gene set enrichment analysis (GSEA) reveals significantly enriched MAPK signaling pathways in LSS downstream target genes. **D** qPCR validation results of MAPK signaling pathway enriched genes. **E** Kyoto Encyclopedia of genes and genomes (KEGG) analysis showed that LSS downregulated gene enrichment pathway after overexpression. **F** qPCR validation results of steroid biosynthesis signaling pathway enriched genes. **G**, **H** Western blotting analysis showing the expression of JNK, p-JNK, P38, p-P38, Erk, and p-Erk after LSS knockdown and overexpression in EC cells. *p < 0.05, **p < 0.01, ***p < 0.001.

To elucidate molecular mechanisms underlying LSS-mediated EC cell growth, we performed RNA sequencing (RNA-seq) to compare the gene expression profiles of LSS-overexpressing Ishikawa cells with control cells. RNA-seq analysis identified 609 upregulated and 349 downregulated genes following LSS overexpression (Fig. 4A). KEGG pathway analysis revealed significant enrichment of the MAPK signaling pathway among the upregulated genes (Fig. 4B, C). qPCR validation confirmed that key MAPK pathway genes, such as DDIT3 and FOS, were significantly upregulated in LSS-overexpressing cells (Fig. 4D). Furthermore, LSS overexpression suppressed the steroid biosynthesis pathway, as indicated by the downregulation of genes like SQLE, DHCR7, and CYP51A1 (Fig. 4E, F). WB analysis confirmed that LSS overexpression increased phosphorylation of P38 and JNK, while LSS knockdown reduced JNK and ERK phosphorylation (Fig. 4G, H). These findings suggest that LSS promotes EC cell growth by activating the MAPK/JNK signaling pathway.

### Lipid metabolism disorders in EC and the role of LSS in cholesterol ester accumulation

**Fig. 5:**
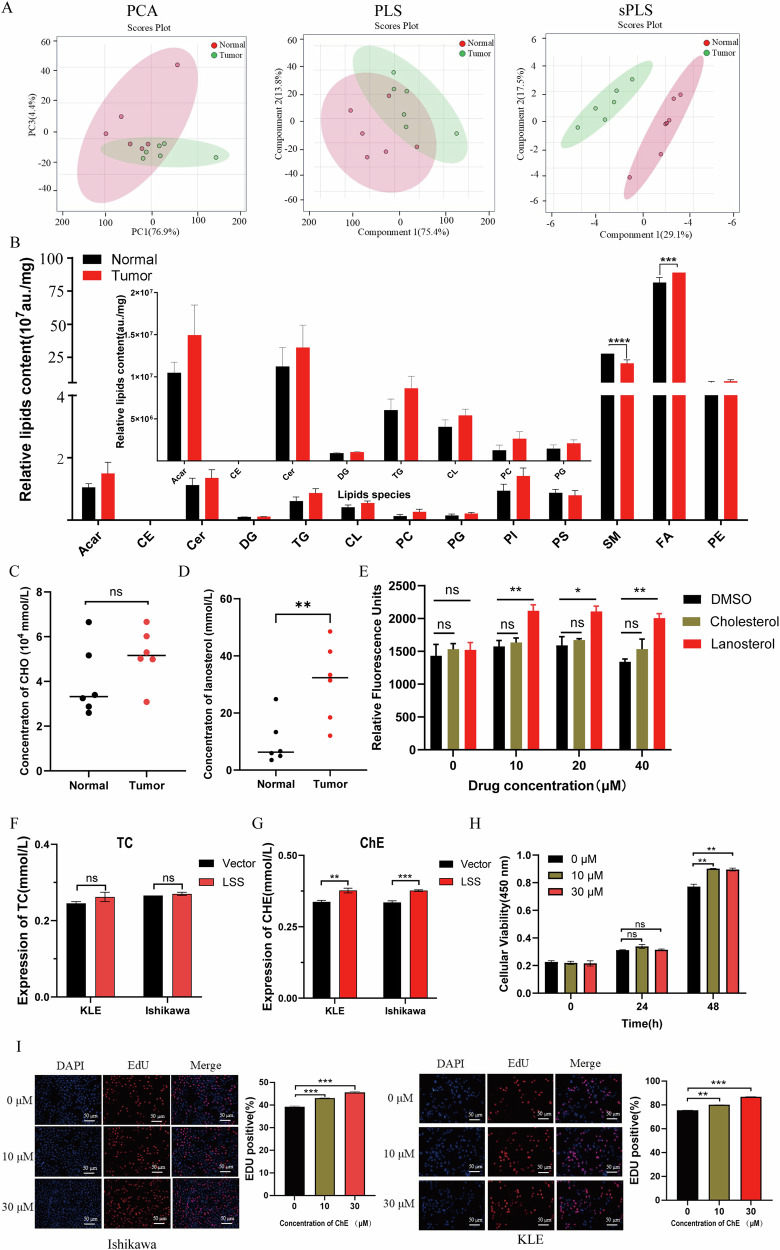
Patients with EC have lipid metabolism disorders, and LSS promotes the growth of EC cells by affecting the accumulation of cholesterol esters. **A** Principal component analysis of lipidomics results in cancer and para-cancer tissues of 6 patients. **B** Statistical graph of expression of various lipids. **C**, **D** Statistics of cholesterol and lanosterol in cancer and para-cancer tissues detected by targeted lipidomics. **E** CCK8 results of exogenous cholesterol and lanosterol on EC cell proliferation. **F**, **G** Results of ELISA detection of total cholesterol (TC) and cholesterol ester (ChE) levels after LSS overexpression. **H** CCK8 results of exogenous cholesterol esters promoting Ishikawa cell proliferation. **I** EdU results of exogenous cholesterol esters promoting EC cell growth. *p < 0.05, **p < 0.01, ***p < 0.001.

Given the involvement of LSS in cholesterol biosynthesis, we investigated lipid metabolism in EC patients using both untargeted and targeted lipidomics. The analysis revealed significant alterations in lipid metabolism in EC tissues, with elevated levels of fatty acids (FA) and cholesterol esters (ChE), and decreased levels of sphingomyelin (SM) (Fig. 5A, B). Targeted lipidomics further showed that lanosterol levels were significantly increased in EC tissues, while total cholesterol levels remained unchanged (Fig. 5C, D). In vitro, the addition of exogenous lanosterol significantly promoted the proliferation of Ishikawa cells (Fig. 5E). ELISA assays confirmed that while total cholesterol levels were unchanged, cholesterol ester levels were significantly elevated following LSS overexpression (Fig. 5F, G). Exogenous cholesterol esters also enhanced the growth and EdU positivity of EC cells (Fig. 5H, I). These results suggest that LSS-driven cholesterol ester accumulation plays a critical role in promoting EC cell growth.

### The LSS inhibitor Ro 48-8071 suppresses EC cell proliferation in vitro and tumor growth in vivo

**Fig. 6:**
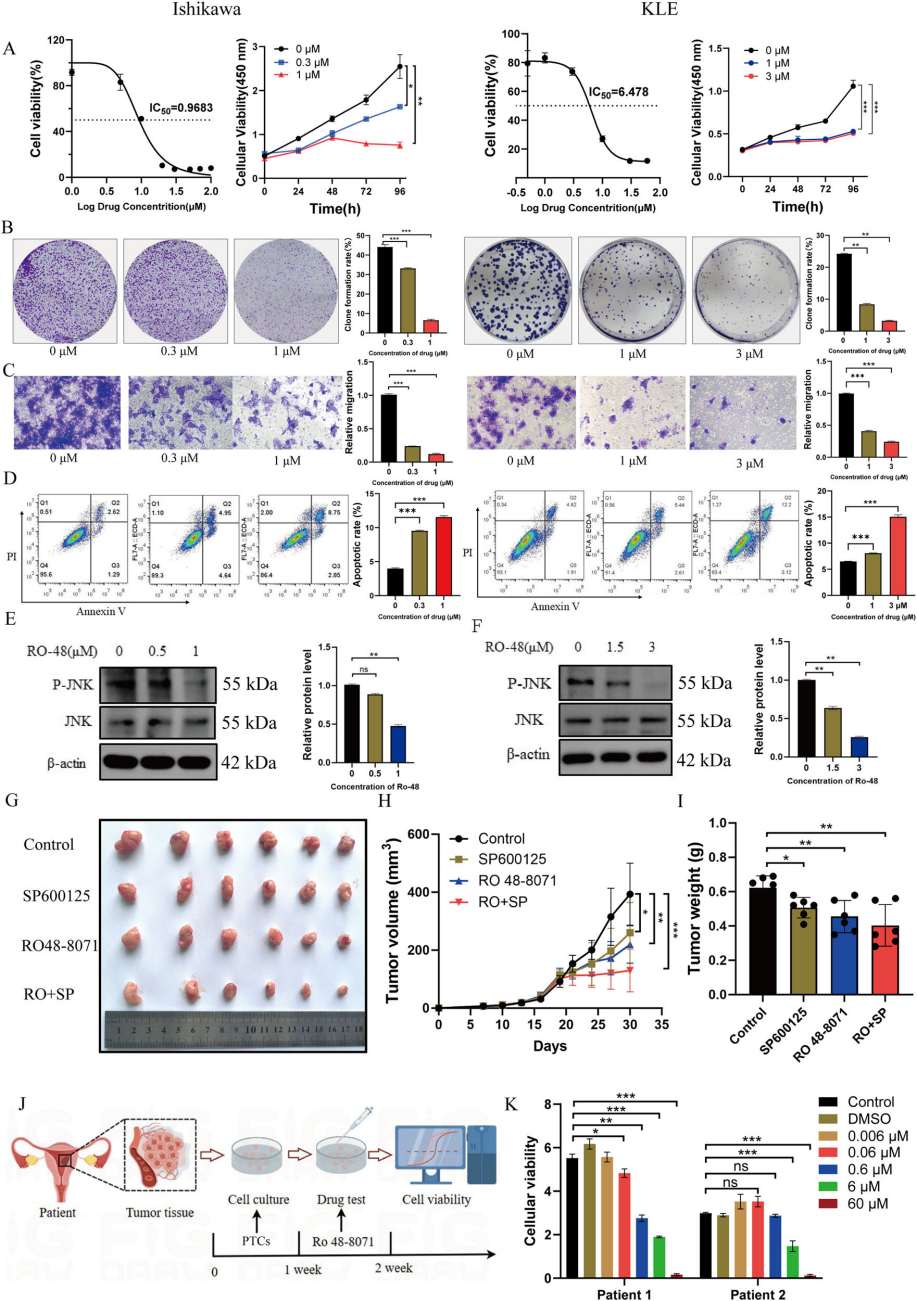
Ro 48-8071 inhibits EC cell proliferation in vitro and tumor growth in vivo. **A** The results of CCK8 reflected the IC_50_ of Ro 48-8071 in EC cells and that Ro 48-8071 significantly inhibited the proliferation of EC cells. **B**, **C** Clone formation and Transwell experiments showed that the activity of EC cells was significantly inhibited by Ro 48-8071. **D** Flow cytometry analysis found that Ro 48-8071 can promote the apoptosis of EC cells. **E**, **F** Ro 48-8071 reduces the expression of phosphorylated JNK protein. **G** Images of isolated tumors from the subcutaneous tumor nude mice model established using Ishikawa cells treated with Ro 48-8071 or SP600125(*n* = 6 mice per group). **H**, **I** Tumor volume and weight in vehicle. **J** Flowcharts of PTCs for the drug-sensitivity test. **K** Viability of PTCs cells after treatment with Ro 48-8071. *p < 0.05; **p < 0.01; ***p < 0.001.

Given the oncogenic role of LSS in EC, we evaluated the therapeutic potential of the LSS inhibitor Ro 48-8071. The IC50 of Ro 48-8071 was determined to be 0.968 μM in Ishikawa cells and 6.478 μM in KLE cells (Fig. 6A). Treatment with Ro 48-8071 significantly inhibited EC cell growth, reduced EdU positivity, and suppressed colony formation (Figs. 6A, B and S4). Ro 48-8071 also inhibited cell migration (Fig. 6C) and induced apoptosis, as evidenced by flow cytometry analysis (Fig. 6D). WB analysis demonstrated that Ro 48-8071 reduced JNK phosphorylation, and co-treatment with the JNK inhibitor SP600125 further enhanced the anti-tumor effects of Ro 48-8071 in vivo (Figs. 6E, F, G–I and S5). To more accurately simulate in vivo conditions, we used the PTCs of EC patients to validate the inhibitory effect of Ro 48-8071 (Fig. S6). The drug-sensitivity test plan is shown in Fig. 6J. We found that Ro 48-8071 inhibited the growth of tumor cells in a concentration-dependent manner in both patient PTCs (Fig. 6K). These results indicate that LSS inhibitor Ro 48-8071 effectively suppresses EC cell proliferation and tumor growth, highlighting its potential as a therapeutic strategy for EC.

## Discussion

In recent years, extensive research has highlighted the critical role of metabolic enzymes in cancer development and progression. Dysregulation of metabolic pathways, including cholesterol metabolism, has been increasingly recognized as a key contributor to tumorigenesis [9, 19, 20]. In endometrial cancer (EC), while the association with estrogen exposure is well-established, the underlying metabolic mechanisms remain poorly understood [21, 22]. Our previous studies have shown that lanosterol synthase (LSS) has the ability to reverse the aggregation of lens proteins and prevent cataracts [23, 24]. This study provides new insights into the role of LSS, a key enzyme in cholesterol biosynthesis, in promoting EC cell proliferation and survival through the activation of the MAPK/JNK signaling pathway and the accumulation of cholesterol esters.

Our findings demonstrate that LSS expression is significantly elevated in EC tissues compared to normal endometrial tissues, with a clear correlation between LSS levels and tumor malignancy (Fig. 1). The upregulation of LSS not only enhances EC cell proliferation and migration but also inhibits apoptosis by activating the MAPK/JNK signaling pathway, thereby contributing to tumor growth both in vitro and in vivo (Figs. 2–4). These results align with previous studies in other cancer types, where LSS has been implicated in promoting oncogenic signaling pathways, such as mTOR and MAPK, and in facilitating tumor progression [25].

MAPK cascade reaction is a key signaling component in regulating various biological processes such as cell differentiation, proliferation, migration, and invasion. The MAPK cascade typically involves four major classical pathways: MEK/ERK, c-Jun nh2 terminal kinase (JNK), p38 MAPK, and ERK5 [26]. Our RNA-seq analysis revealed that LSS overexpression significantly upregulates MAPK pathway genes, particularly those involved in the JNK and P38 branches, which are known to be activated in response to cellular stress and are often associated with cancer progression (Fig. 4C-H). Numerous studies have indicated that the MAPK signaling pathway plays a pivotal role in the onset and progression of EC, and interfering with this pathway can effectively hinder the advancement of EC diseases [27, 28]. Additionally, pharmacological agents targeting this pathway have shown potential as therapeutic interventions for EC [29]. Interestingly, we observed that LSS overexpression led to the suppression of the steroid biosynthesis pathway, including the downregulation of *SQLE*, *DHCR7*, and *CYP51A1*, suggesting a complex interplay between cholesterol biosynthesis and MAPK signaling in EC. In our prior research, we identified that SQLE, the gene directly upstream of LSS in the cholesterol biosynthesis pathway, is markedly upregulated in EC tissues and facilitates the growth of EC cells via activation of the PI3K/AKT signaling pathway [27–30]. A Mendelian randomization study also discovered an association between abnormal cholesterol levels in blood lipids and the development of EC [22]. Furthermore, cholesterol-lowering drugs have been found to inhibit the growth of EC cells [31–33]. This connection between cholesterol metabolism and signaling pathways underscores the multifaceted role of lipids in cancer biology.

One of the most striking findings of this study is the role of LSS in modulating lipid metabolism, particularly in the accumulation of cholesterol esters. Lipidomics analysis revealed significant alterations in lipid profiles in EC tissues, with increased levels of fatty acids and cholesterol esters (Fig. 5), which are known to support the rapid proliferation and survival of cancer cells [34–36]. The accumulation of cholesterol esters has been implicated in the progression of several cancers, including colorectal, liver, and pancreatic cancers [37, 38]. In EC, we found that LSS overexpression drives the accumulation of these esters, further promoting tumor growth. This highlights the potential of targeting cholesterol esterification as a therapeutic strategy in EC.

The therapeutic potential of LSS inhibition was further supported by our in vitro and in vivo experiments with Ro 48-8071, a specific LSS inhibitor (Fig. 6). Ro 48-8071 effectively suppressed EC cell proliferation, induced apoptosis, and inhibited tumor growth in a nude mouse model. Numerous studies have validated its effectiveness in inhibiting tumor growth, including breast cancer, prostate cancer, and liver cancer [17, 18]. Notably, the combination of Ro 48-8071 with the JNK inhibitor SP600125 enhanced its anti-tumor efficacy, suggesting a synergistic effect between LSS inhibition and MAPK pathway blockade. Furthermore, in drug treatment experiments using patient-derived endometrial cancer organoids, Ro 48-8071 was also found to significantly inhibit organoid growth, underscoring its potential as a therapeutic strategy for EC. These findings underscore the promise of LSS as a therapeutic target in EC, with Ro 48-8071 representing a potential lead compound for further development.

While this study provides significant insights into the role of LSS in EC, it also raises several questions that warrant further investigation. The exact mechanisms by which LSS modulates cholesterol ester accumulation and its impact on tumor metabolism require deeper exploration. Additionally, the observed interplay between LSS and MAPK signaling suggests that other signaling pathways may be involved in LSS-mediated tumorigenesis. Future studies should aim to elucidate these mechanisms and assess the broader implications of LSS inhibition in EC, including its effects on metastasis and resistance to therapy.

**Fig. 7:**
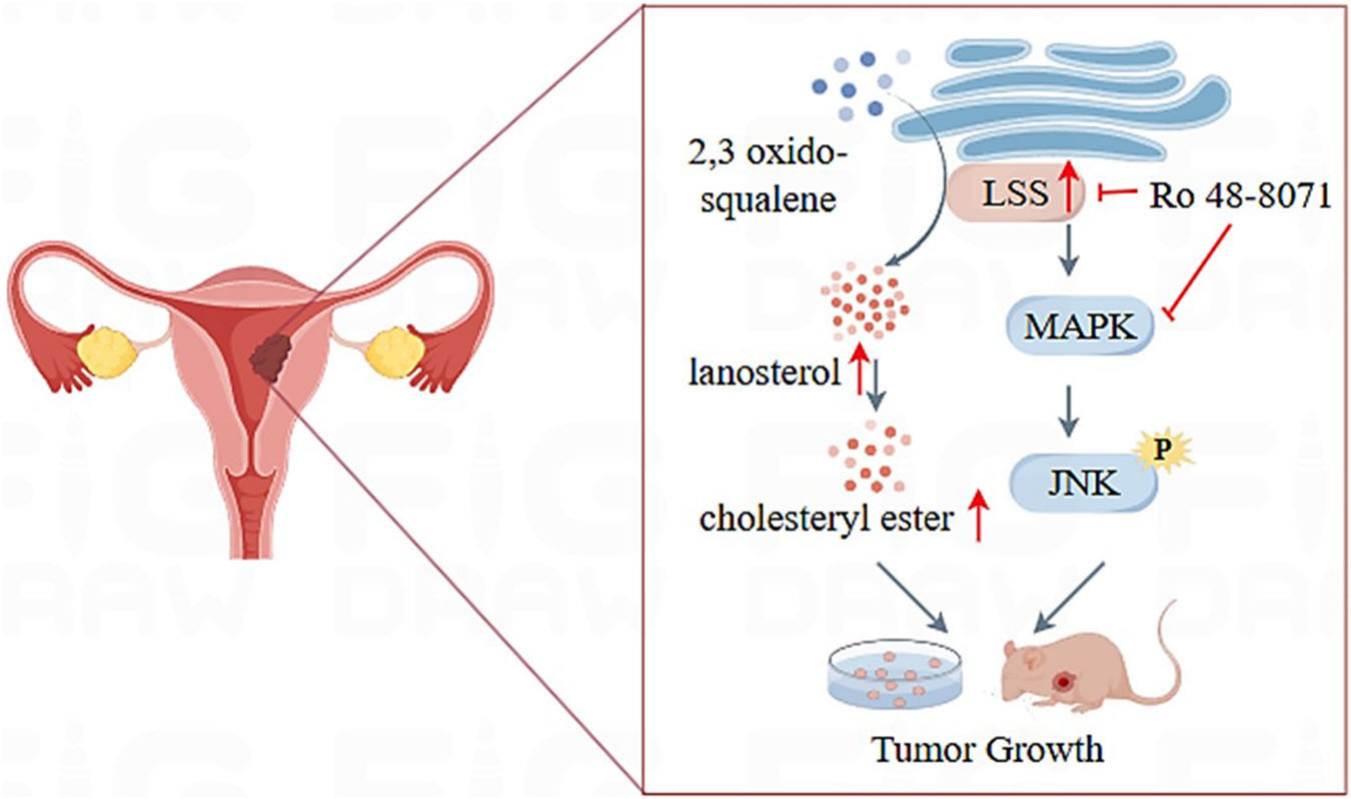
Schematic of the function and mechanism of LSS in EC (red arrows indicate increased expression).

In conclusion, our study identifies LSS as a key driver of EC progression through its dual role in activating the MAPK/JNK signaling pathway and promoting cholesterol ester accumulation (Fig. 7). Targeting LSS, either alone or in combination with MAPK pathway inhibitors, offers a promising therapeutic approach for the treatment of EC. Further research is needed to fully understand the potential of LSS inhibitors in clinical settings and to explore their efficacy in combination with other targeted therapies.

## Conclusions

This study demonstrates that LSS is a key regulator of cholesterol metabolism, driving endometrial cancer (EC) cell growth and tumorigenesis. The oncogenic effects of LSS are mediated through the activation of the MAPK/JNK signaling pathway and the promotion of cholesterol ester accumulation. Importantly, the LSS inhibitor Ro 48-8071 significantly inhibited EC growth in both in vitro *and* in vivo models, as well as in patient-derived organoids, highlighting LSS as a promising therapeutic target for EC.

## Materials and methods

### Clinical samples

Paired cancer tissue and adjacent endometrial tissue (*n* = 8) were taken from patients who underwent surgery at Lanzhou University First Hospital (LDYYLL2023- 184; LDYYLL2024-76). All specimens obtained informed consent from patients and were approved by the hospital’s ethics committee. None of the patients received local or systemic treatment before surgery.

### Western blotting

The tissue is rapidly frozen and crushed in liquid nitrogen. Protein extraction was performed on tissue powder and cells using RIPA lysate containing a mixture of protease and phosphatase inhibitors (Beyotime Biotechnology, Shanghai, China). Perform SDS-PAGE on an equal amount of protein, transfer it onto a nitrocellulose filter membrane, and then incubate overnight with specific primary antibodies (Table S1). The membranes were Visualized by enhancing the chemiluminescence detection kit (Thermo Scientific, Waltham, MA, USA).

### RNA extraction and quantitative real-time PCR analysis

According to the manufacturer’s plan, use the RNAkey total RNA extraction kit to extract total RNA from tissues or cells. Perform reverse transcription on PrimeScriptTMRT Master Mix (Takara, Beijing, China). Perform qPCR reaction using EvaGreen Dye (Biotium, Fremont, USA) or Hieff qPCR SYBR Green Master Mix on MX3005P circulator (Agilent, Santa Clara, CA, USA). Measure relative mRNA levels using the comparative Ct method and normalize by β - actin mRNA levels. LSS (primers: forward 5′-GGCAGTGGGACCTACCT-3′ and reverse 5′-GAAAGTGGGCCACCATATATC-3′) and β-actin (primers: forward 5′-GTCGACGAGCG-3′ and reverse 5′-GAACAG AGCCCCCTT-3′).

### Cell culture and reagents

The Ishikawa and KLE cell lines were purchased from the China Center of American Type Culture Collection (ATCC, Wuhan, China) and cultured in the DMEM or DMEM/F12-Dulbecco’s Modified Eagle Medium (Servicebio) with 10% Fetal bovine serum (FBS, Gibco) at 37 °C with 5% CO_2_. Chemical compounds: Ro 48-8071(#HY-18630A) and SP600125(#HY-12041) from MedChemExpress; Cholesteryl elaidate (#19485-76-8) from Macklin; Cholesterol (#HY-N0322) from MedChemExpress.

### Immunohistochemistry (IHC)

Detailed immunohistochemical (IHC) analysis was performed using standard methods. The primary antibodies used include anti-LSS (# 13715-1-AP), Proteintech,1:200; anti-Ki67 (#GB151499-100, Servicebio,1:500). Calculate the percentage of positively stained cells in the total number of tumor cells using Image J software.

### Plasmids and stable cell lines construction

After 24 h of cell adhesion in a 6-well plate, plvx-IRES-LSS and shLSS lentivirus were added (ObiO Technology, Shanghai, China). After processing the cells for 24 h, replace the culture medium. After 72 h of virus introduction, cells were observed under a microscope to confirm the successful transduction of lentivirus into target cells. After 3 days of treatment with puromycin (Gibco, Shanghai, China), the transfection efficiency was detected using Western blotting (WB).

### Cell growth assessment

To evaluate cell growth, 5 × 10^3^ EC cells were inoculated into a 96-well plate. Add 10 μL Cell Count Kit-8 (CCK8, # G1613-5ML, Servicebio) at appropriate time intervals and incubate at 37 °C for 1 h. Measure the absorbance at 450 nm using a Microplate reader.

### EdU cell proliferation assay

To evaluate the proliferation ability of EC cells, BeyoClick with Alexa Fluor 488 (# KTA2031, Abbkine) was used according to the manufacturer’s instructions (working solution 10 μM) ™ The EdU cell proliferation assay kit was used to determine the proliferation of EdU cells. Use fluorescence microscopy to observe and determine the ratio of EdU-positive cells using Image J.

### Colony formation assay

Conduct colony formation measurements to evaluate the size and quantity of the colonies. Inoculate cells (1–3 × 10^3^ cells/well, depending on cell type) into a 6-well plate. After 7–10 days of treatment with different concentrations of drugs, remove the culture medium and wash the cells three times with PBS. Colonies were fixed with 4% paraformaldehyde and stained with 0.5% crystal violet. Count only colonies with more than 50 cells per colony.

### Migration assay

To observe cell migration ability, a Transwell chamber (Costa, NY, USA; 8 mm pore size) was used for migration determination in a 24-well culture dish. Cells were seeded into a small chamber at a rate of 3 × 10^4^ cells per well and then incubated at 37 °C in 5% CO_2_ for 48 h. Select 3 random fields for each membrane for photography and count them using an inverted phase contrast microscope.

### Apoptosis assay

To determine cell apoptosis, cells were seeded into a 6-well plate and collected after overexpression, knockdown, or drug treatment. Under strict light avoidance conditions, dyes were added and the apoptotic ability of cells was detected by flow cytometry. Simultaneously, WB was used to detect the expression changes of apoptosis-related proteins BAX, BCL-2, and Caspase-3.

### Cellular ACE inhibitory assay

To determine the cytotoxicity of Ro 48-8071 on KLE and Ishikawa cells. Dissolve Ro 48-8071 in water to prepare a 10 mM solution. Inoculate 5 × 10^3^ cells onto a 96-well plate. After adhering to the wall for 24 h, replace 100 µL of fresh culture medium containing Ro 48-8071, which is continuously diluted at a concentration of 1 to 20 μM. After 48 h of cultivation, carefully remove the culture medium and add 100 µL of newly prepared CCK8 solution (10 µL CCK8 pre-mixed with 90 µL complete culture medium) to each well. After incubation for 1 h, measure the absorbance of each well at 450 nm using an enzyme-linked immunosorbent assay (ELISA) reader. The IC50 value is calculated as the concentration of each compound required to inhibit 50% cell growth relative to the compound-free control (GraphPad Prism 8.0).

### Measurement of cholesterol levels

Collect cells (1 × 10^6^) and use a cholesterol ester quantification kit (#CB10478 Hu, Coibo Bio, shanghai), The cholesterol quantification test kit (# PMK1143) measures total cholesterol and cholesterol ester levels.

### Animal experiments

The Ncr nu/nu nude mice were provided by GemPharmatech (Jiangsu, China) Experimental Animal Company. All experiments were conducted with the approval of the Animal Ethics Committee of Lanzhou University First Hospital. The mice were randomly assigned after 1 week of adaptive feeding. Subcutaneously inject 5 × 10^6^ cells into the armpits of 6-week-old female Ncr nu/nu nude mice (*n* = 4 in overexpression group; *n* = 6 in drug group). To investigate the effects of inhibitors Ro 48-8071 and SP600125, nude mice were orally administered daily (RO 20 mg/kg; SP 10 mg/kg). Tumor size was monitored using a digital caliper, and the tumor volume was calculated using the formula [sagittal dimension (mm) × transverse dimension (mm)^2^]/2, expressed in mm^3^. When the study concluded, the mice were euthanized, and the tumors were collected for analysis.

### Culture of EC PTCs

Collect 200 mg of fresh endometrial cancer tumor tissue and store the collected fresh samples in pre-cooled PBS (10 mM HEPES and 100 U/mL penicillin–streptomycin) (Thermo Fisher Scientific). Remove as much necrotic area and fatty tissue as possible. The tissue was cut into small pieces and digested in PBS/EDTA 1 mM containing 200 U/ml collagenase I (Thermo Fisher Scientific) for 1 h. Free cells were collected using a 40 mm filter. After centrifugation for 10 minutes (300 × *g*, 4 °C), the cell microspheres were resuspended in PTC growth medium and seeded at 105 cells/cm^2^. The cells were cultured in a 37 °C, 5% CO_2_ incubator. PTC growth medium was refreshed every 2–3 days, as necessary. The completion of this work was assisted by GeneX (Zhejiang) Precision Medicine Co., Ltd.

### RNA-seq analysis

Extract total RNA from Vector or LSS Ishikawa cells (*n* = 3 per group). RNA sequencing (RNA seq) was performed on the Illumina HiSeq 2500 platform. Use the HISAT2 algorithm to map sequencing readings to the human genome GRCh38 reference genome. We conducted differential expression analysis between the Vector and LSS Ishikawa groups using the DESeq2 R package (|Fold Change| > 1.5, padj <0.05). The heatmap was created using the pheatmap R package. Finally, using the Kyoto Encyclopedia of Genes and Genomes (KEGG) database and clusterPro ® R package enriches differentially expressed genes through pathways.

### Targeted metabolomics analysis

Collect 20 mg of cancer tissue and pair adjacent cancer tissue for targeted metabolomics analysis. Extract total lipids from tissues and use cholesterol and lanosterol standards as a reference to detect the content of cholesterol and lanosterol in cancer tissues. The testing services are provided by the Pharmaceutical Technology Center of Tsinghua University.

### Bioinformatic analysis

We downloaded microarray datasets (GSE339099) that were obtained from the Gene Expression Omnibus (GEO; http://www.ncbi.nlm.nih.gov/geo/). The GraphPad Prism 8 was used for statistical analysis and graphing.

### Statistical analysis

GraphPad Prism 8 was used for statistical analysis. Data are represented as the mean ± standard error of the mean (SEM) of three independent experiments. Differences between groups were assessed through a one-way analysis of variance. A *p*-value of <0.05 was considered statistically significant.

## Supplementary information

Supplementary material

Original western blots

## Data Availability

All the data and experimental details in this article may be obtained from the corresponding author upon reasonable request.
