# Supplementary material for: Inhibition of lanosterol synthase linking with MAPK/JNK signaling pathway suppresses endometrial cancer
Source: Cell Death Discov. 2025 Feb 8;11:55. doi: 10.1038/s41420-025-02325-y (PMC11807098; doi:10.1038/s41420-025-02325-y)

**Supplementary Table 1** Details of antibodies

| Antibodies | company | Cat# | Dilution |
| --- | --- | --- | --- |
| LSS | proteintech | 13715-1-AP | 1:1000 |
| JNK | upingBio | YP-Ab-14804 | 1:1000 |
| P-JNK | upingBio | YP-Ab-17797 | 1:750 |
| Caspsae3 | upingBio | YP-Ab-00004 | 1:1000 |
| P38 | CST | 8690 | 1:1000 |
| p-p38 | CST | 4511 | 1:1000 |
| Erk | CST | 4695 | 1:1000 |
| p-erk | CST | 4370 | 1:2000 |
| Bcl-2 | abclonal | A0208 | 1:1000 |
| Bax | abclonal | 50599-2 | 1:1000 |
| Ki67 | Servicebio | GB121141-100 | 1;300 |

**Supplementary Table 2 clinical information of the patients**


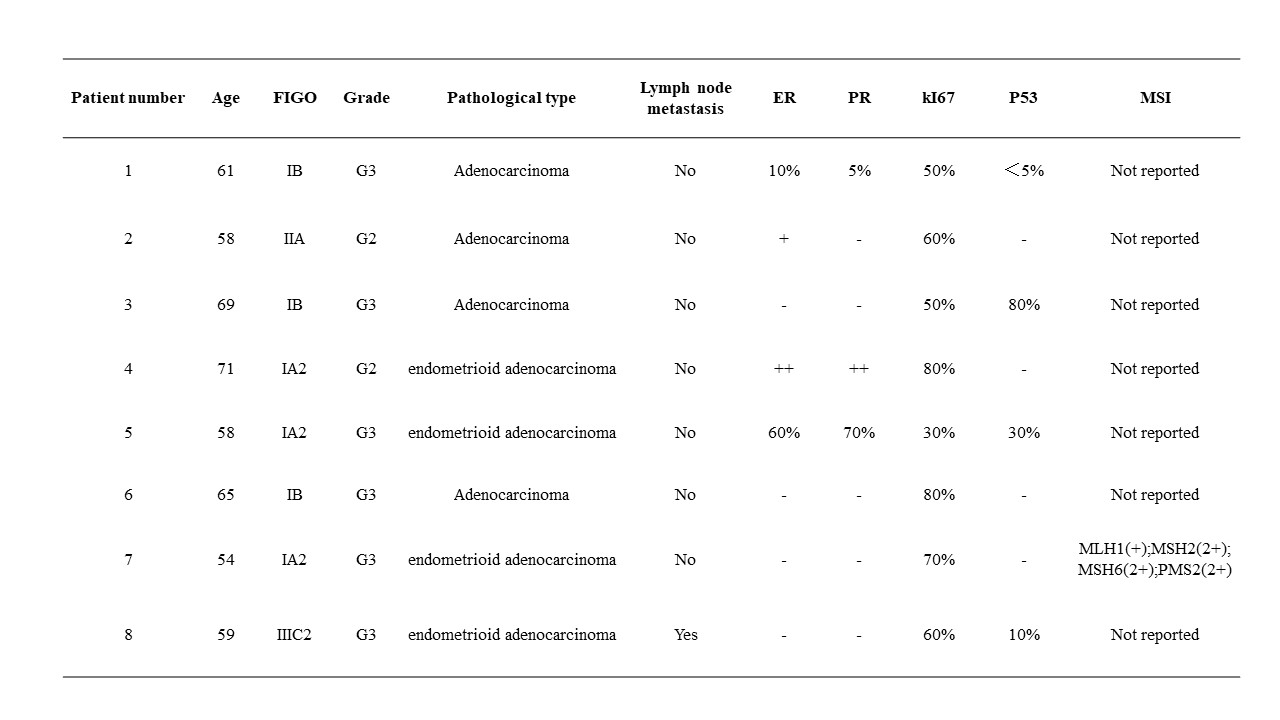


**Supplementary Table 3 shRNA sequence of LSS**


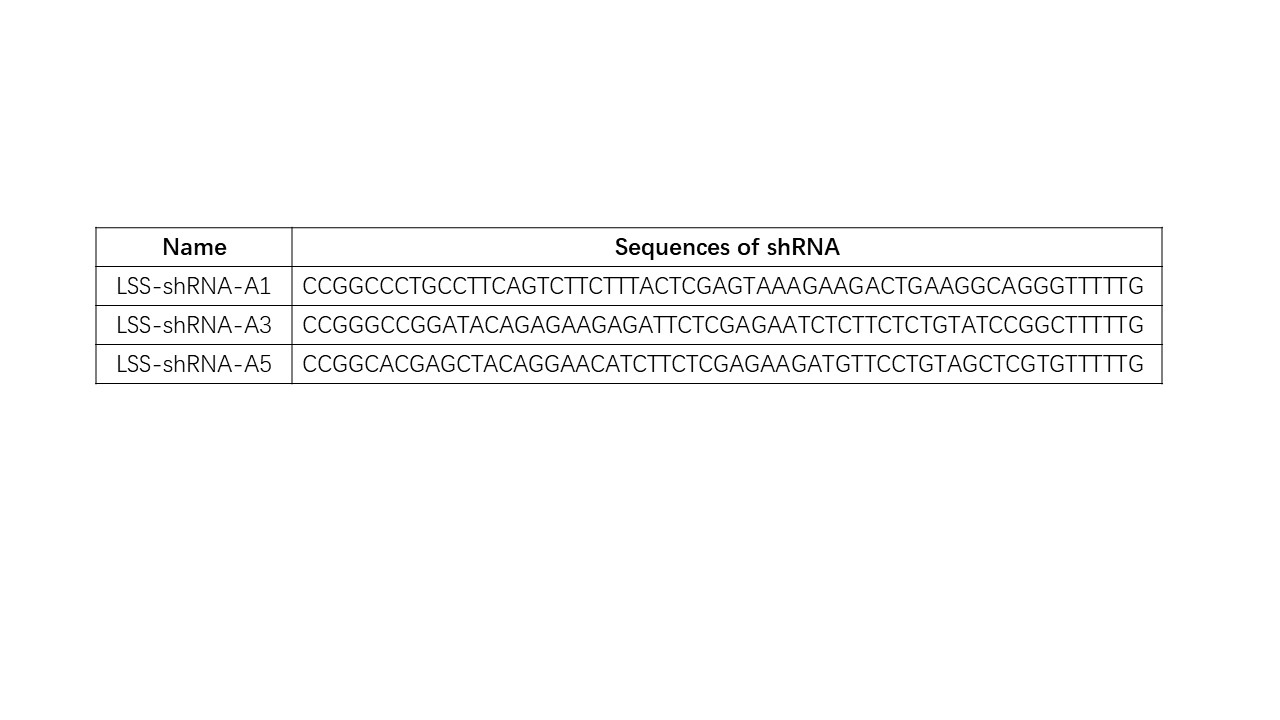


**Supplementary Fig 1 Expression of LSS in normal endometrial cells and cancer cells**


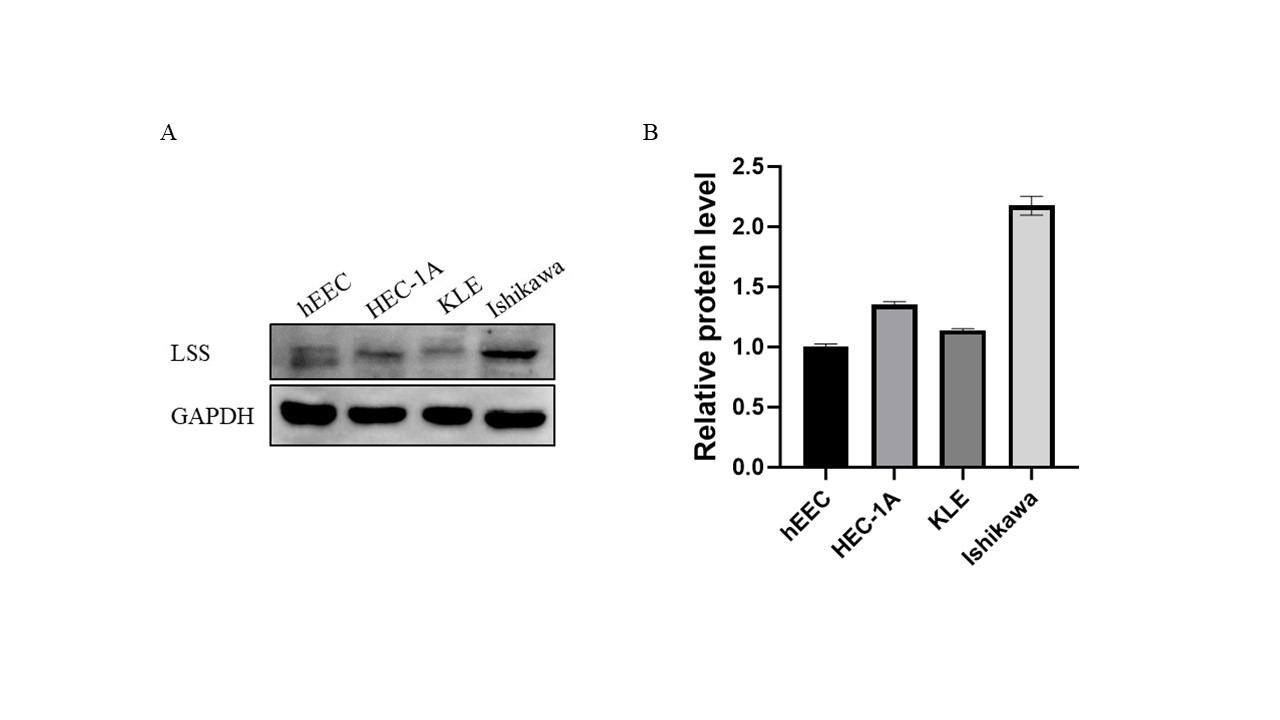


**Supplementary Fig 2 Immunohistochemical images of LSS overexpressed tumors**


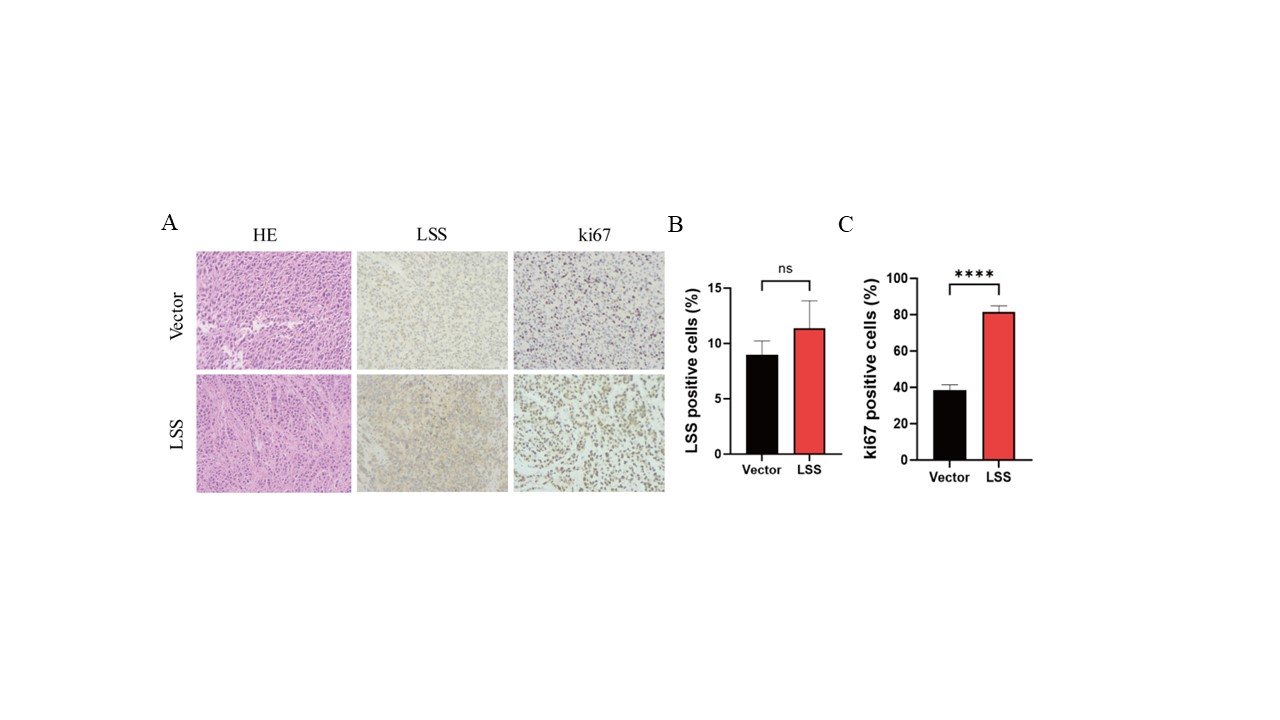


**Supplementary Fig 3 verification of shRNA knockdown**


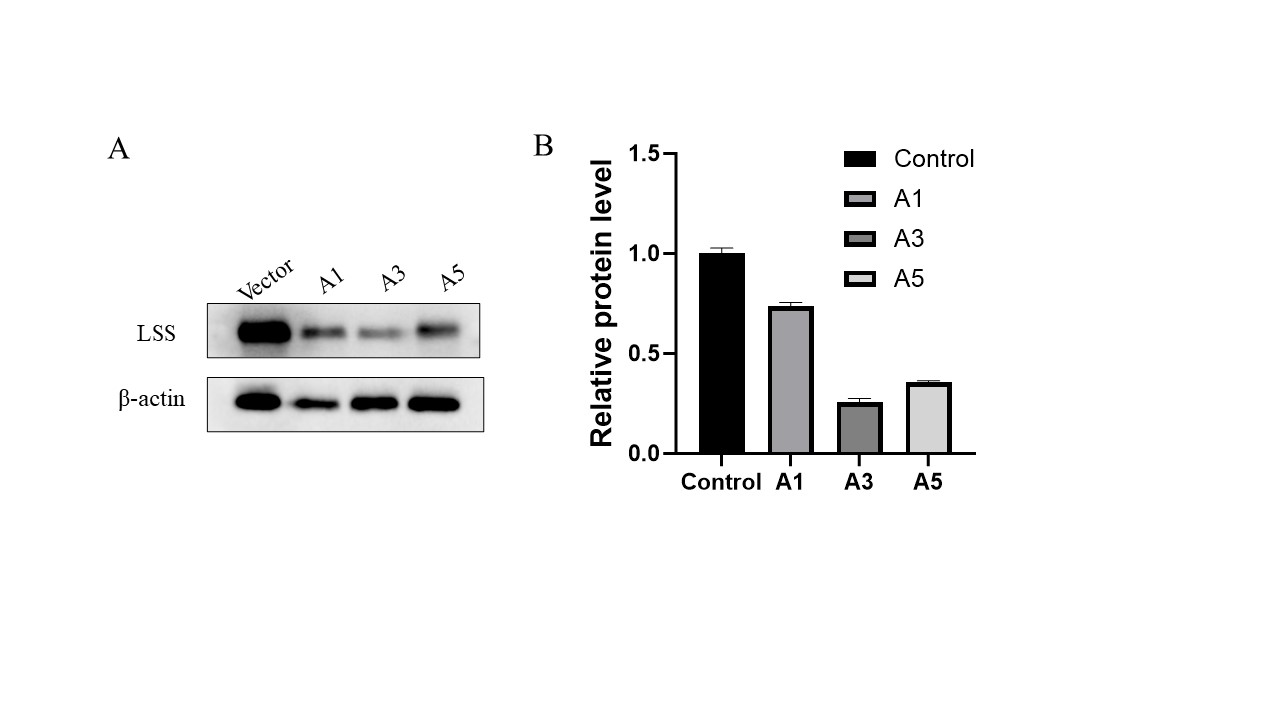


**Supplementary Fig 4 The combination of RO 48-8071 and SP600125 significantly inhibits EC cell migration and colony formation**


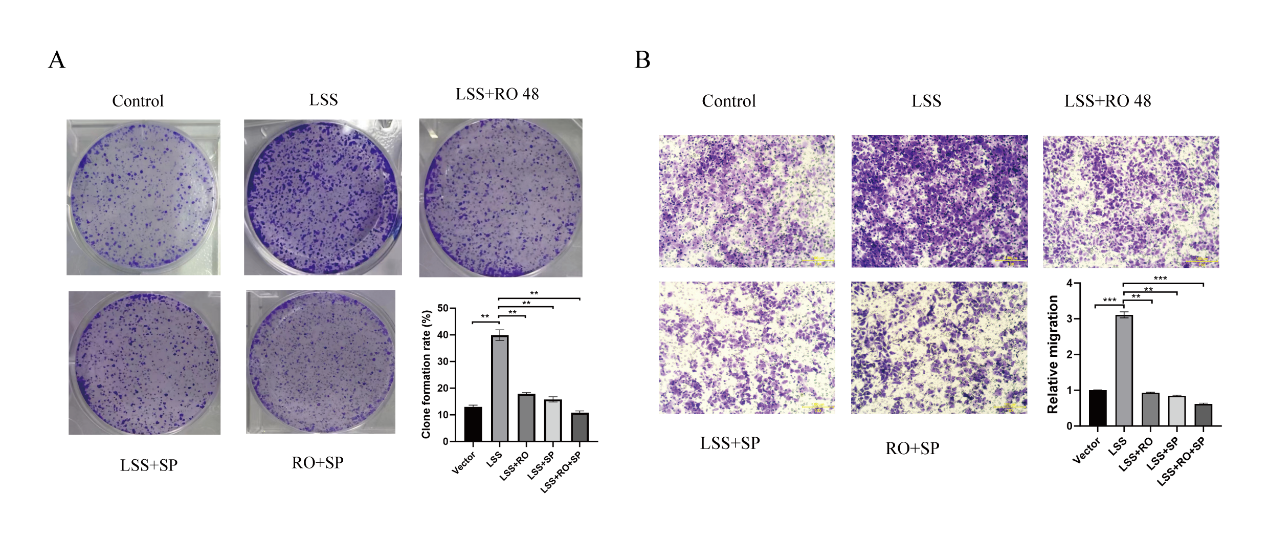


**Supplementary Fig 5 Expression of Ki67 and LSS proteins in mouse tumors**


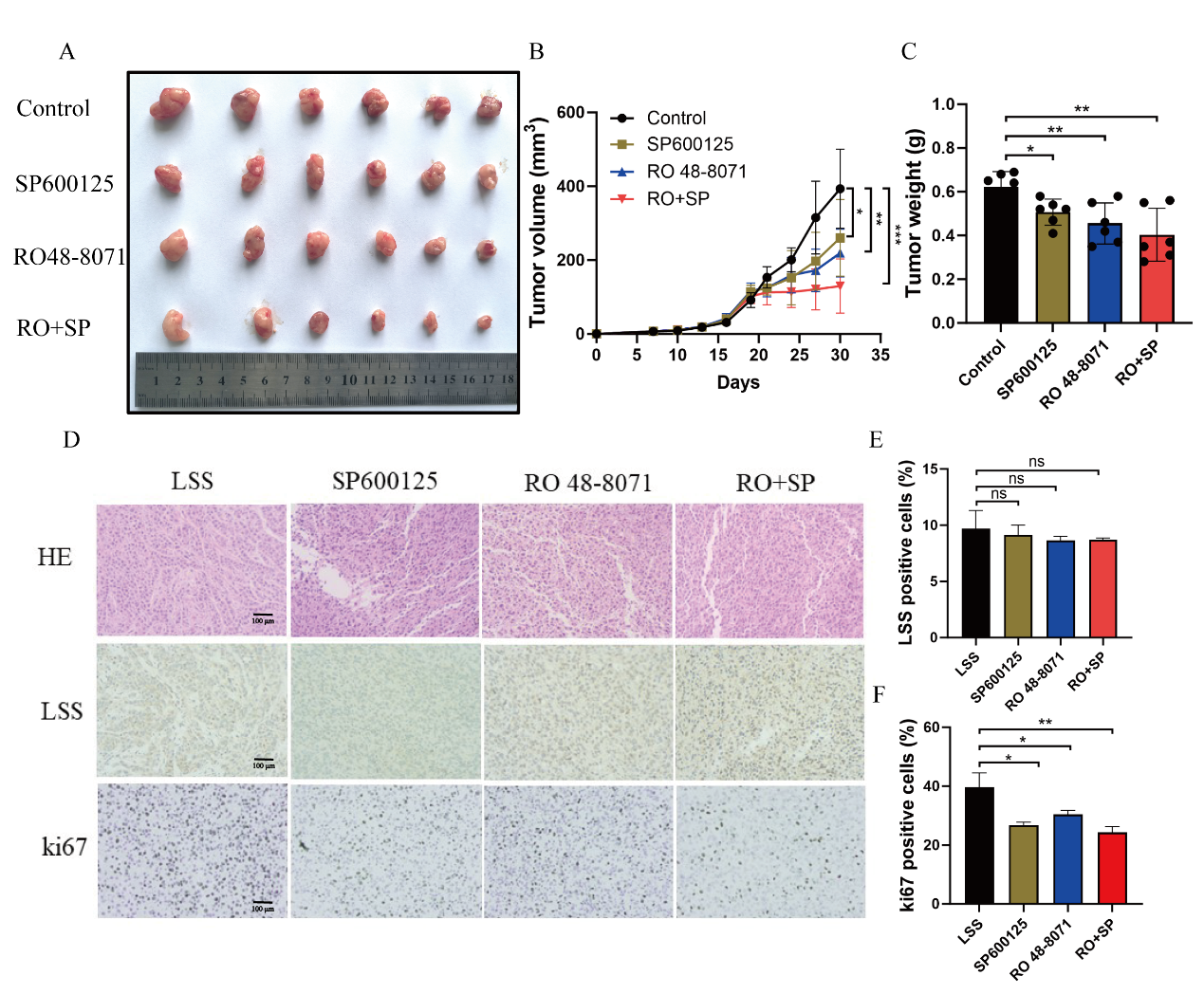


**Supplementary Fig 6 Image of patient derived tumor-like cell cluster culture**


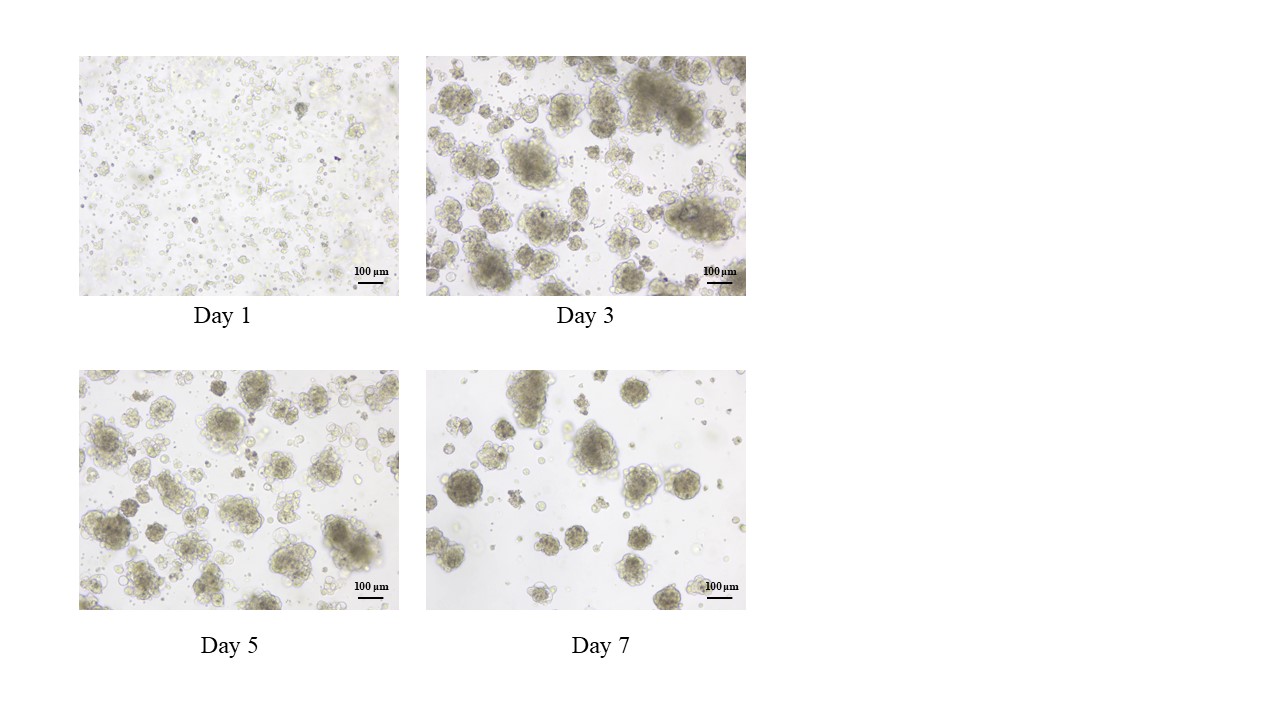

Supplement: Supplementary file 1 — Supplementary material [file 41420_2025_2325_MOESM1_ESM.docx]
