# Supplementary material for: Inhibition of lanosterol synthase linking with MAPK/JNK signaling pathway suppresses endometrial cancer
Source: Cell Death Discov. 2025 Feb 8;11:55. doi: 10.1038/s41420-025-02325-y (PMC11807098; doi:10.1038/s41420-025-02325-y)
Supplement: Supplementary file 2 — Original western blots [file 41420_2025_2325_MOESM2_ESM.pptx]

## Slide 1
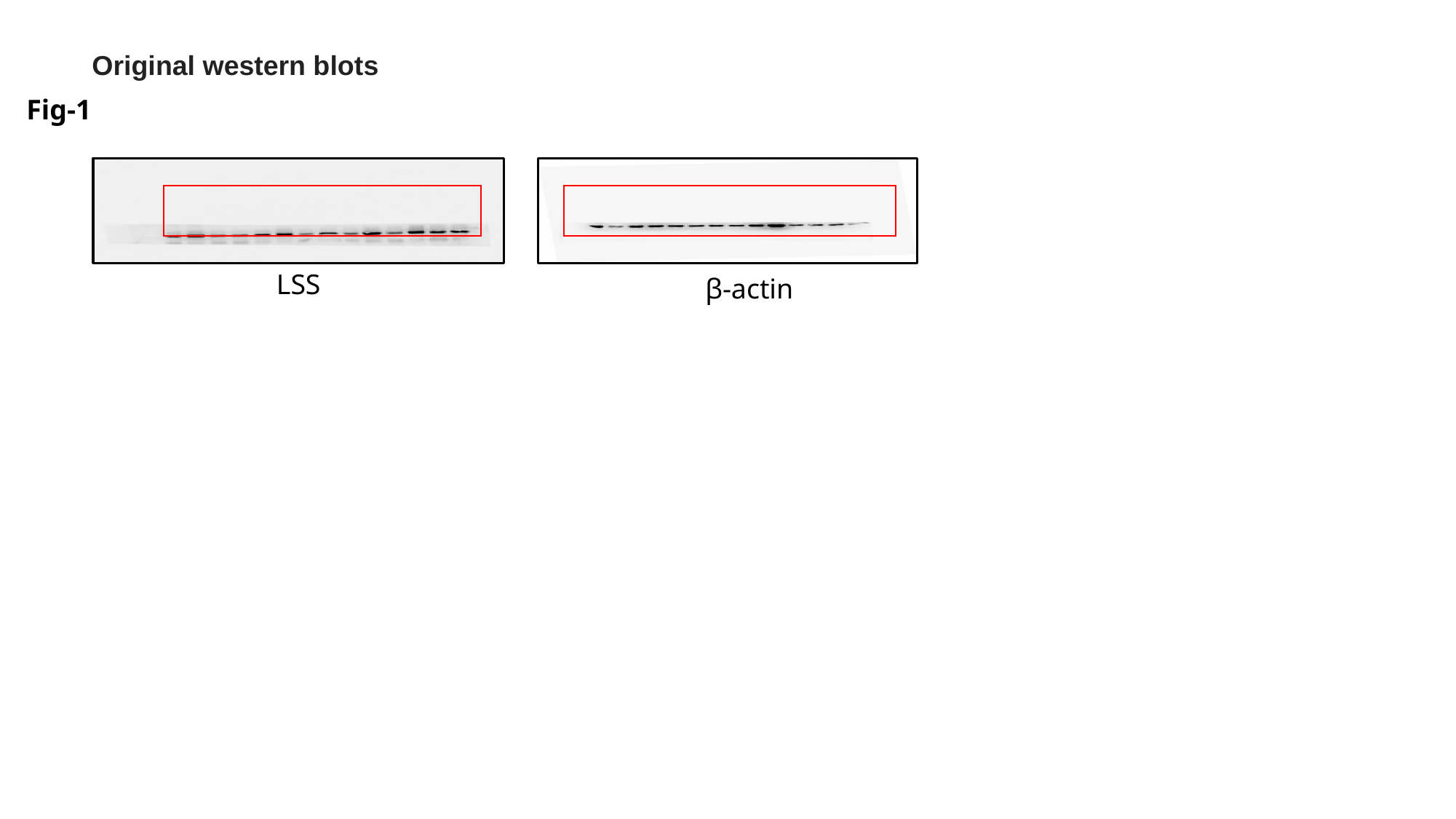

Original western blots
Fig-1
LSS
β-actin

## Slide 2
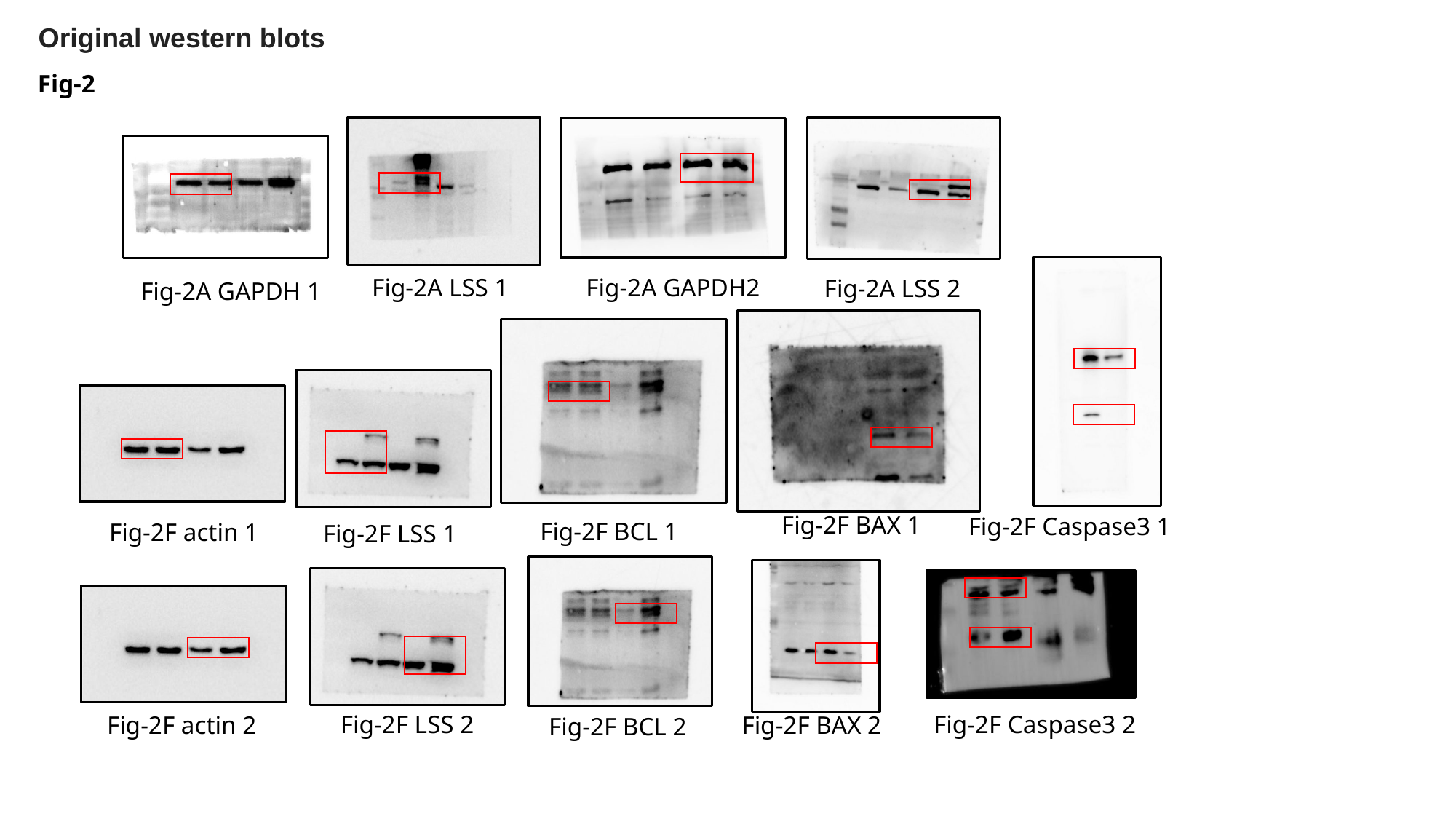

Original western blots
Fig-2
Fig-2A LSS 1
Fig-2A LSS 2
Fig-2A GAPDH 1
Fig-2F Caspase3 1
Fig-2A GAPDH2
Fig-2F BAX 1
Fig-2F BCL 1
Fig-2F actin 1
Fig-2F LSS 1
Fig-2F BCL 2
Fig-2F BAX 2
Fig-2F actin 2
Fig-2F LSS 2
Fig-2F Caspase3 2

## Slide 3
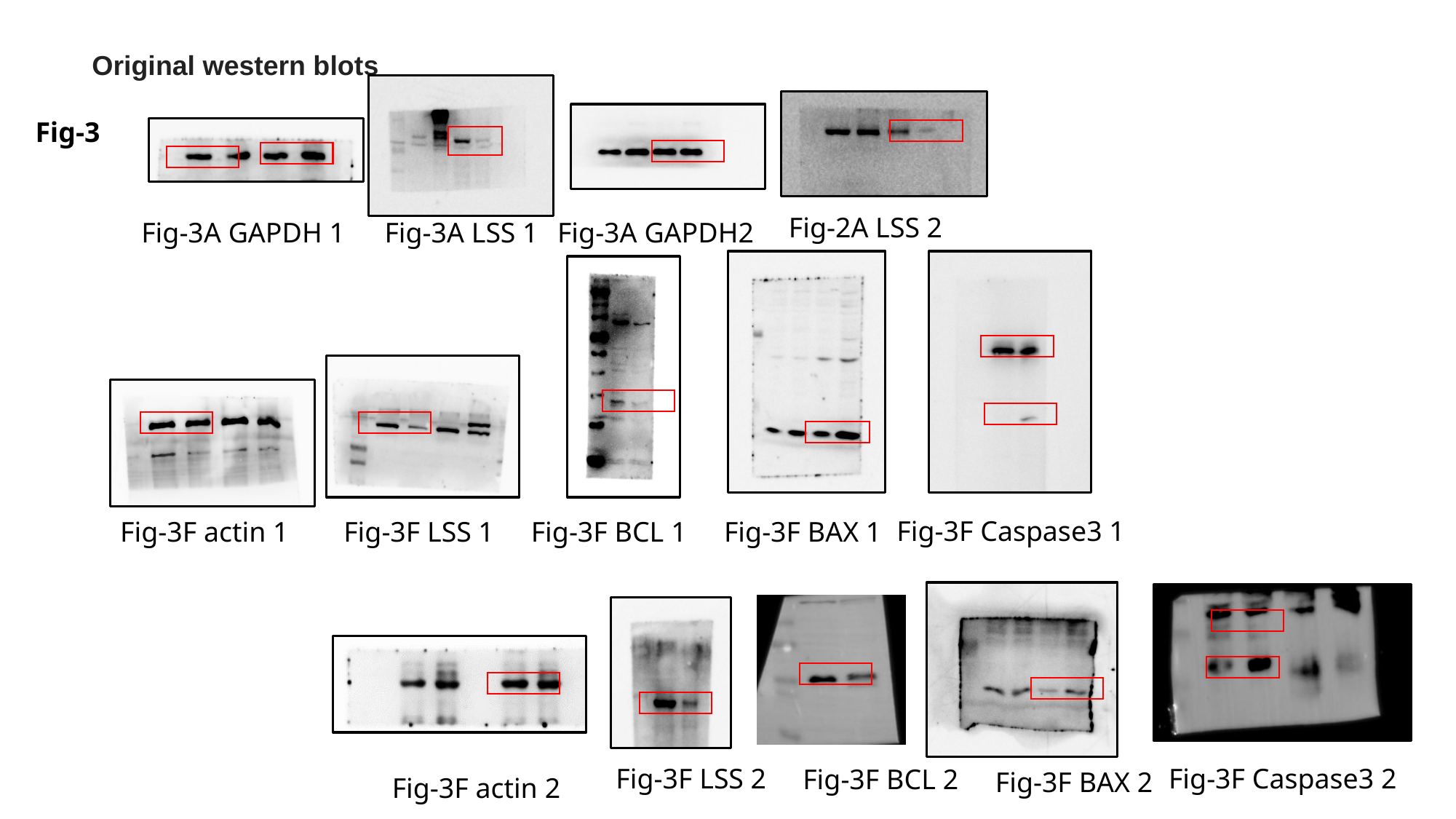

Original western blots
Fig-3
Fig-2A LSS 2
Fig-3A GAPDH 1
Fig-3A LSS 1
Fig-3A GAPDH2
Fig-3F Caspase3 1
Fig-3F actin 1
Fig-3F LSS 1
Fig-3F BCL 1
Fig-3F BAX 1
Fig-3F LSS 2
Fig-3F Caspase3 2
Fig-3F BCL 2
Fig-3F BAX 2
Fig-3F actin 2

## Slide 4
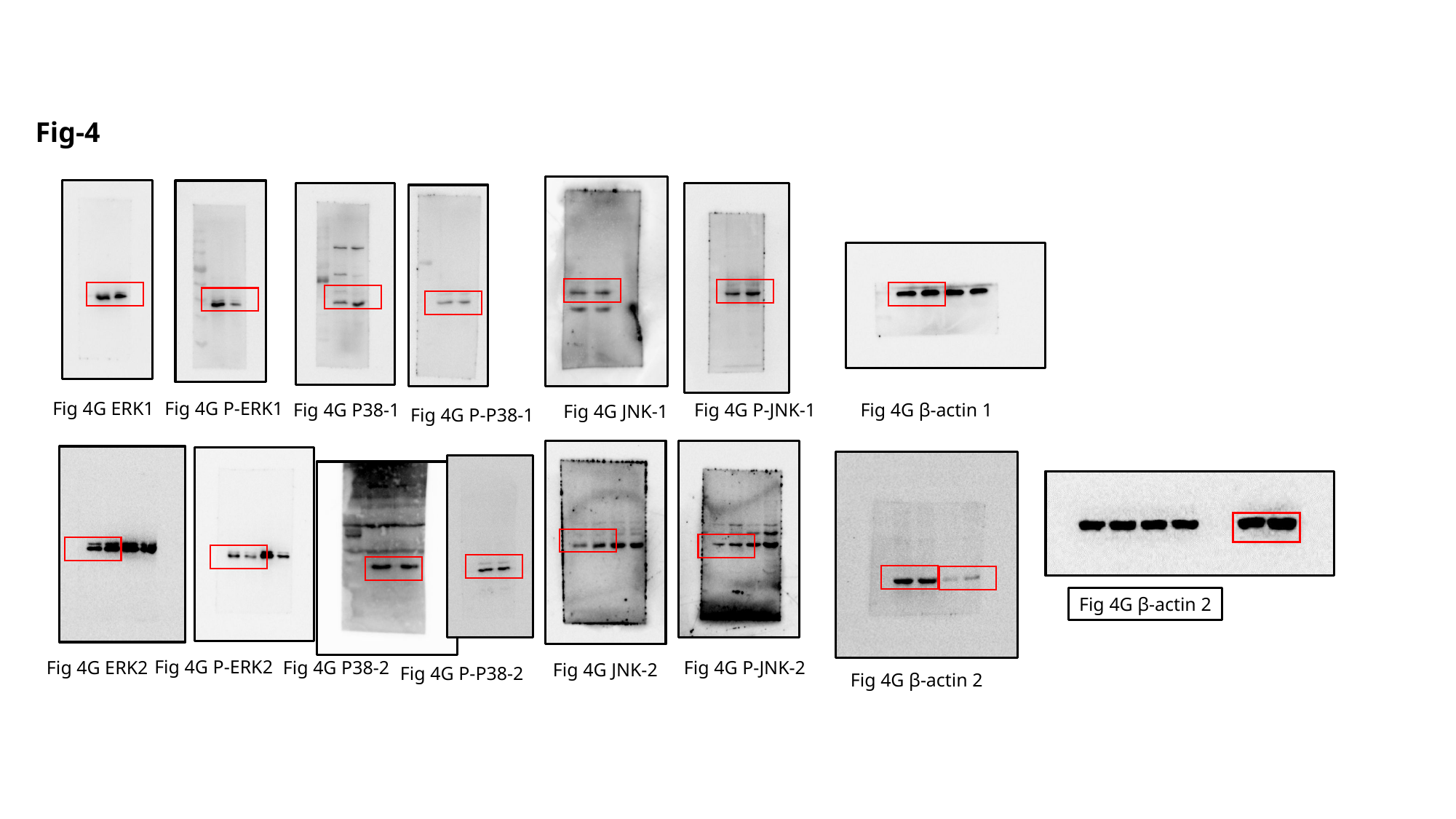

Fig-4
Fig 4G ERK1
Fig 4G P-ERK1
Fig 4G P-JNK-1
Fig 4G P38-1
Fig 4G β-actin 1
Fig 4G JNK-1
Fig 4G P-P38-1
Fig 4G β-actin 2
Fig 4G P-ERK2
Fig 4G ERK2
Fig 4G P-JNK-2
Fig 4G P38-2
Fig 4G JNK-2
Fig 4G P-P38-2
Fig 4G β-actin 2

## Slide 5
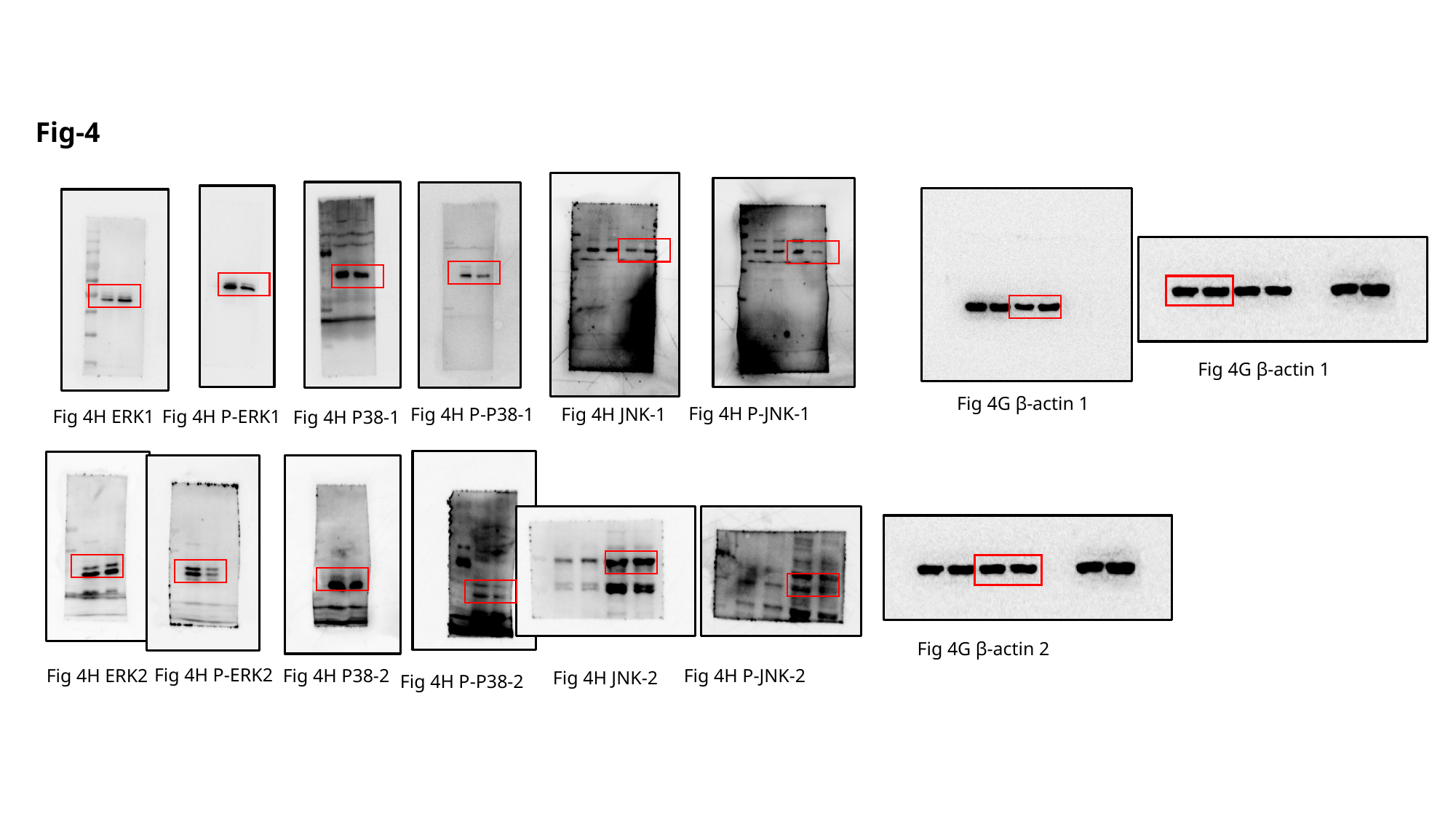

Fig-4
Fig 4G β-actin 1
Fig 4G β-actin 1
Fig 4H P-JNK-1
Fig 4H P-P38-1
Fig 4H JNK-1
Fig 4H ERK1
Fig 4H P-ERK1
Fig 4H P38-1
Fig 4G β-actin 2
Fig 4H P-ERK2
Fig 4H ERK2
Fig 4H P-JNK-2
Fig 4H P38-2
Fig 4H JNK-2
Fig 4H P-P38-2

## Slide 6
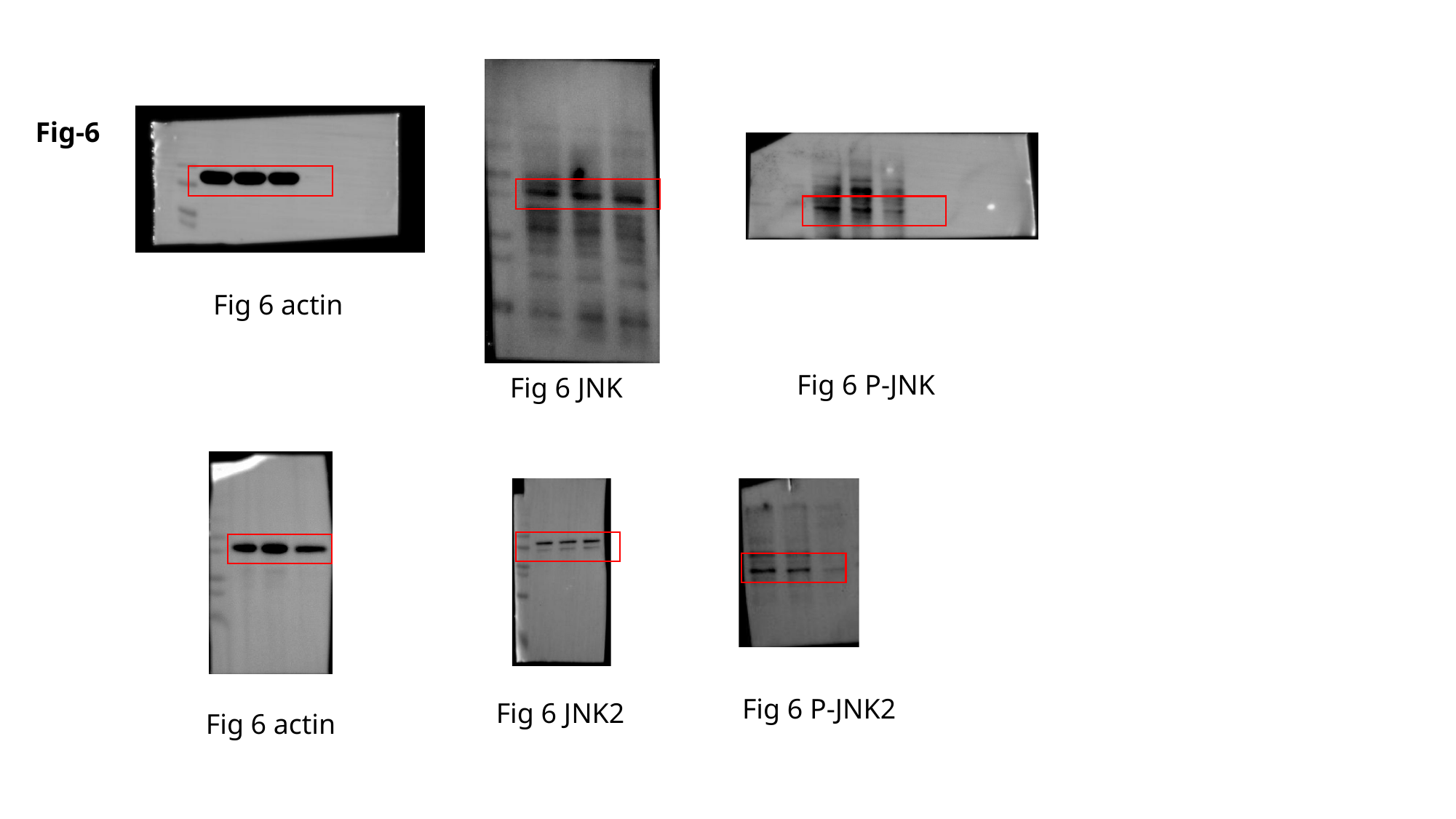

Fig-6
Fig 6 actin
Fig 6 P-JNK
Fig 6 JNK
Fig 6 P-JNK2
Fig 6 JNK2
Fig 6 actin
